# Nondegradative Synthetic Molecular Glues Enter the Clinic

**DOI:** 10.1002/cmdc.202500048

**Published:** 2025-04-14

**Authors:** Maximilian L. Repity, Robin C. E. Deutscher, Felix Hausch

**Affiliations:** ^1^ Department Chemistry and Biochemistry Clemens‐Schöpf‐Institute Technical University Darmstadt Peter‐Grünberg Strasse 4 64287 Darmstadt Germany; ^2^ Centre for Synthetic Biology Technical University 64287 Darmstadt Germany

**Keywords:** covalent warheads, cyclophilin, KRas, macrocycles, molecular glues

## Abstract

Molecular glues are small molecules that can induce or stabilize protein–protein interactions between proteins inside cells. Unlike classical small molecule drugs, molecular glues can target challenging disease‐causing proteins lacking well‐defined binding pockets. Nature has repeatedly used this mode of action, but identifying molecular glues for new target proteins has been a major challenge. Recently, manmade molecular glues, inspired by natural products, for KRas, entered clinical trials although KRas is a major cancer target long thought to be undruggable. Here, how these molecules are initially discovered and optimized to provide several advanced drug candidates for various KRas‐dependent cancer types are outlined. The major insights obtained for this new class of drug modalities are further summarized. These results showcase how molecular glues that do not rely on protein degradation can provide clinical benefits for challenging drug targets.

## Graphical abstract

Proximity‐inducing drugs are a novel therapeutic modality and currently mainly focused on target degradation. Recently, nondegradative molecular glues, inspired by natural products that reprogram cyclophilin A (Cyp A) to inhibit K‐Ras, have entered the clinic. This concept reconstructs the development of drug candidates by the company Revolution Medicines and points toward future opportunities for nondegradative molecular glues.

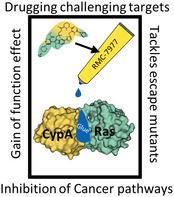

## 1 Introductions

Molecular glues are small molecules that can chemically induce proximity between two proteins.^[^ ^1^ ^]^ This can be pharmacologically used in cells to block undesired protein–protein interactions (competitive molecular glues),^[^ ^2^ ^]^ strengthen existing ones (protein‐protein stabilizers),^[^ ^3^ ^]^ or degrade target proteins, if one of the protein partners is an E3 ligase (molecular glue degraders).^[^ ^4^ ^]^ Often, one of the protein partners of molecular glues can be described as the target protein that is the primary actor of the observed or desired pharmacological effect. The other protein partner can then be described as an accessory partner or presenter protein which is critical for enabling the molecular glue and its special pharmacological properties. A major advantage of molecular glues is that they do not necessarily require well‐defined binding pockets on the target protein to interact with it. Molecular glues compensate for this by utilizing chemically induced protein–protein interactions to enhance binding to the target. This opens the prospect of improving the druggability of the human proteome and suggests exciting and so far unprecedented opportunities for refined and possibly context‐specific or gain‐of‐function pharmacology.

Nature has repeatedly used the molecular glue concept as evidenced by natural products such as rapamycin (FKBP12‐rapamycin‐FRB complex),^[^ ^5^, ^6^, ^7^, ^8^ ^]^ FK506 (FKBP12‐FK506‐calcineurin complex),^[^ ^9^, ^10^ ^]^ WDB002 (FKBP12‐WDB002‐CEP250 complex),^[^ ^11^ ^]^ cyclophilin A (cyclophlin A‐cyclosporin A‐calcineurin complex),^[^ ^12^, ^13^ ^]^ sanglifehrin A (cyclophlin A‐sanglifehrin A‐IMPDH2 complex),^[^ ^14^, ^15^ ^]^ antascomicin B (FKBP51‐AntaB‐ Akt1 complex),^[^ ^16^, ^17^ ^]^ or fusicoccin A (H(+)‐ATPase‐fusicoccin A‐14‐3‐3 proteins).^[^ ^18^ ^]^ These natural products all induce ternary complexes with fundamentally different target proteins. Inspired by this and based on pioneering work by Stuart Schreiber, chemical biologists routinely used analogs of FK506 or rapamycin to chemically induce dimerization of engineered proteins inside cells.^[^ ^19^ ^]^ Other prime examples of molecular glues are immunomodulatory imide drugs (IMiDs) such as thalidomide, which induce ternary complexes with the E3 ligase Cereblon and targeted degradation of various target proteins.^[^ ^4^ ^]^

Discovering novel molecular glues from scratch has so far been challenging, and most known (nondegradative) molecular glues have been discovered by serendipity. However, stimulated by the success of heterobifunctional proteasomal degraders (known best under their brand name: PROTACs) and IMiDs, strategies are emerging to discover synthetic molecular glues more systematically.^[^ ^20^, ^21^ ^]^ Recent examples include DNA‐encoded glue screening,^[^ ^22^, ^23^, ^24^ ^]^ assays with adaptor proteins at high concentrations,^[^ ^25^ ^]^ CRISPR screenings that can be accompanied by luminescence‐based readouts such as the HiBit system,^[^ ^26^ ^]^ and proteomics‐based target identification.^[^ ^27^ ^]^ In addition, RIPTACs, a class of heterobifunctional molecules pursued by Halda Therapeutics, have emerged.^[^ ^28^ ^]^ These modalities aim the leverage disease‐specific expression of presenter proteins to enable cell type‐specific target protein inhibition without degrading the latter. Recently, the first RIPTACs have entered the clinic.^[^ ^29^ ^]^

The small GTPase KRas has been one of the most promising but also most challenging proteins to drug for the last decades. KRas and the homologous NRas and HRas are mutated in 10–30% of all cancers.^[^ ^30^ ^]^ Mutations in Ras proteins lead to their continuous activation by stabilizing the so‐called “switch” regions in the protein in their active conformation. This drives uncontrolled cell proliferation, for example, via the RAF‐MEK‐ERK pathway, the Ral‐GEF pathway, and the PI3K‐AKT‐mTOR pathway.^[^ ^31^ ^]^ For instance, KRas^G12C^ is common in lung cancer and KRas^G12D^ in colorectal cancer and pancreatic cancer.^[^ ^30^ ^]^ All KRas isoforms have a conserved GTP‐binding pocket that could be theoretically targeted with conventional inhibitors. However, direct active‐site inhibitors were unsuccessful due to the picomolar affinity for the endogenous substrate GTP.^[^ ^32^ ^]^ Since 2013, multiple transient‐binding pockets have been discovered in Ras, which triggered the discovery of a variety of Ras inhibitors with selectivity for specific mutants, such as G12C and G12D to pan‐selective KRas inhibitors and KRas degraders.^[^ ^33^, ^34^ ^]^ A prominent initial hit for KRas was discovered by a tethering strategy with the Ras mutation KRas^G12C^^[^ ^35^ ^]^ that led to the development of several inhibitors that bind and trap KRas in its inactive state.^[^ ^34^ ^]^ Several of these Ras inhibitors have been approved or are currently being examined in clinical trials.^[^ ^36^ ^]^ Despite these compounds representing a large milestone towards a druggable K‐Ras, their response rates are limited with 37% for sorotasib^[^ ^37^ ^]^ and 43% for adagrasib.^[^ ^38^ ^]^ A Phase 3 clinical study comparing sotorasib with the proved chemotherapy drug docetaxel reveals that for sotorasib, progression‐free survival was improved, and side effects were less severe than for Docetaxel, but the differences in overall survival were only marginal.^[^ ^39^ ^]^ This demonstrates that there is still room for improvement in the development of novel Ras inhibitors. Here, we review the development of KRas‐targeting molecular glues by the biotech company Revolution Medicines based on pioneering work by the company Warp Drive Bio.

## 2 The Early Days: From Natural Product Cyclophilin Ligands to the First KRas Glues

**Figure 1:**
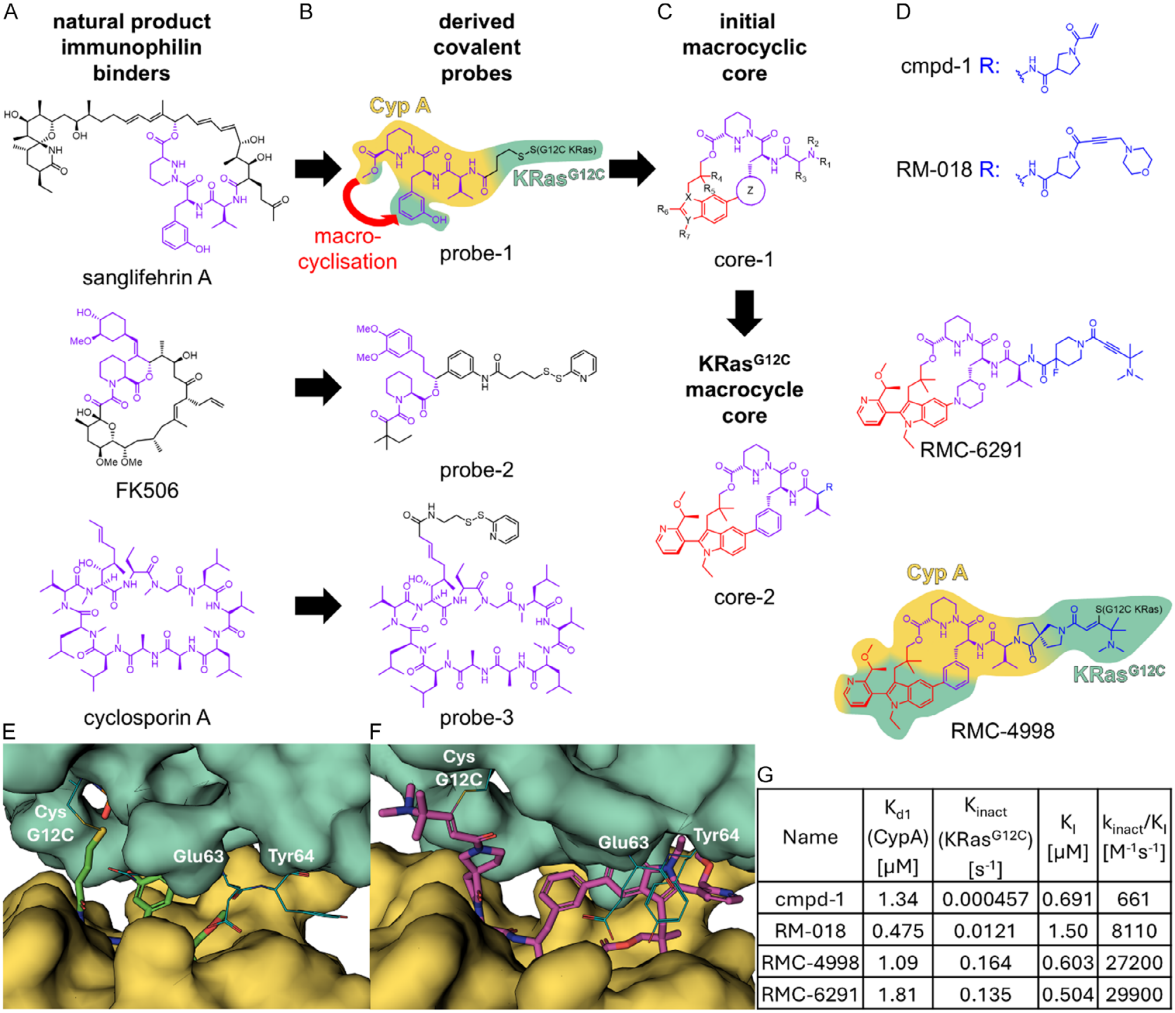
Development of RMC‐6291. A) Natural product immunophilin binders (sanglifehrin A, FK506, and cyclosporin A), where CypA/FKBP‐binding parts are highlighted in purple. B) Synthetic analogs derived from the natural products shown in (A), which contain a covalent warhead; for probe‐1, the primary CypA and KRas‐binding parts are shadowed yellow and cyan, respectively. C) The sanglifehrin A‐based covalent probe (probe‐1) was further optimized through macrocyclization of the initial macrocyclic core (core‐1), which was the basis for all advanced macrocyclic KRas^G12C^ inhibitors (core‐2). The parts of the molecules that were added for the ring closure of the macrocycle are highlighted in red, and parts kept the same as in sanglifehrin A are shown in purple. D) KRas^G12C^‐targeting compounds cmpd‐1, RM‐018, RMC‐4998 (shadowed based on CypA/KRasG12C interactions as for probe‐1), and RMC‐6291^[^ ^42^ ^]^ were derived from core‐2 by elaboration of the linker part (marked in blue) connecting macrocycle core (core‐2) to the warhead. E,F) Ternary complexes of cyclophilin A (yellow surface), KRas^G12C^ (cyan surface with amino acids in cyan lines for better visibility), and probe‐1 (E) green sticks, covalently bound to KRas^G12C^, PDB‐ID 8g9q) or RMC‐4998 (F) magenta sticks, bound to KRasG12C, PDB‐ID 8g9p). G) Table with binding and inactivation constants for the shown inhibitors.

When KRas was still considered undruggable, molecular glues offered an alternative approach for drugging KRas that did not rely on druggable pockets of KRas. With this in mind, the biotech company Warp Drive Bio started a molecular glue program that focused on glueing KRas^G12C^ to the adaptor proteins cyclophilin A (CypA) or FKBP12, two prominent members of the immunophilin family. CypA and FKBP12 were chosen because several natural product molecular glues had proven these proteins to be suitable presenter proteins.^[^ ^1^, ^40^ ^]^ Moreover, the immunosuppressants cyclosporin A, FK506, and rapamycin have validated CypA‐ or FKBP12‐based molecular glue approaches to be clinically viable. KRas^G12C^ presented an ideal target for the chemically induced proximity approach as it allowed to establish the trimeric complex by a covalent bond between a warhead on a CypA/FKBP binder and G12C of KRas. To this end, several probes were designed^[^ ^41^ ^]^ that were derived from known natural ligands for cyclophilins (cyclosporin A and sanglifehrin A) and FKBPs (FK506) (**Figure** **1**A). Three representative probes from patent US20170097359 are exemplarily shown in Figure 1B. For the initial probes, a cysteine‐reactive reversible covalent warhead was added to the natural products or to synthetically derived CypA/FKBP‐binding core scaffolds. Attachment points were chosen to avoid interference with CypA/FKBP binding based on the known binding modes of the CypA/FKBP ligands. The length of the linkers connecting to the warheads was varied. The covalent mode of action allowed for highly sensitive readouts to detect the formation of the ternary complex, incl. mass spectrometry, western blot, and size exclusion chromatography. This in turn was crucial to identify the first weak molecular glue hits, which were further characterized by crystal structures of the ternary complexes. From these initial hits,^[^ ^41^ ^]^ the sanglifehrin A‐derived probe‐1 was further advanced at least in part due to more promising drug‐like properties.

## 3 The Development of RMC‐6291

The analysis of the cocrystal structure of probe‐1 in the ternary complex with CypA and KRas^G12C^ (Figure 1E) revealed direct protein–protein interactions between cyclophilin A and KRas^G12C^ such as hydrogen bonds formed between Trp^121^ and Asn^149^ of cyclophilin A and Glu^63^, Glu^37^ and Asp^38^ of KRas^G12C^, respectively. Such direct protein–protein interactions are crucial for molecular glues to establish sufficient binding energy for ternary complex formation and are difficult to predict a priori. The discovery of this molecular glue binding mode constituted the basis for further development. Based on the initial structure, several modifications of probe‐1 were made.^[^ ^42^ ^]^

A key step was the macrocyclization between the phenyl ring and the ester. Here, the initial structures consisted of a fused ring system with two rings attached to the phenyl ring in meta position and a linker consisting of three carbon atoms between the ester oxygen and the fused ring system (Figure 1C, core‐1, marked in red).^[^ ^43^ ^]^ For the fused ring system, an indole seemed to be the preferred choice, although the nitrogen was initially located at the X position, instead of the later preferred Y position (core‐1). The macrocyclization likely was based on several rationales. Besides profiting from the general benefits of macrocyclization such as a decrease of conformational entropy, better pharmacodynamic, and pharmacokinetic properties for beyond‐rule‐of‐five compounds,^[^ ^44^ ^]^ an increase of protein contacts, especially with KRas^G12C^, was achieved (compare probe‐1 and RMC‐4998, Figure 1E,F).

To satisfy the requirements of the ternary structure, the attachment point for the macrocyclization was different from the ones chosen for other macrocyclic cyclophilin A inhibitors, where the carboxylic acid ester and the nitrogen of the valine were connected.^[^ ^45^, ^46^, ^47^ ^]^

The late‐stage optimization converged on the macrocyclic structure core‐2, which was used to develop advanced inhibitors targeting KRas^G12C^. For the transition from core‐1 to core‐2, every part of the macrocycle except the piperazine ring was changed. The modifications on the indole ring (*R*_6_ and *R*_7_) were mainly designed to increase the interaction surface with KRas^G12C^, while the methyl substitutions on the carbon chain (*R*_4_ and *R*_5_) were closer to CypA. In the Z‐ring, the hydroxy group was discarded in comparison to probe‐1 to reduce the total number of hydrogen bond donors and acceptors and possibly to reduce glucuronidation, while the valine side chain (*R*_3_) was kept.^[^ ^48^ ^]^

The structure of core‐2 sets the scaffold for further variations on the warhead and the connection to the warhead (*R*). Here, a large number of variations were explored, as suggested by patent disclosures.^[^ ^49^ ^]^ The goal was to reduce the general reactivity of the warhead and to compensate for this by optimal positioning of the warhead in the context of the ternary complex. Therefore, changes were made to enhance *k*_inact_(KRas^G12C^), while the affinity toward CypA (*K*_d1_) or the *K*_I_ toward facilitating the ternary complex remains similar (Figure 1G). Indeed, the *k*_inact_ and, in turn, the *k*_inact_/*K*_I_ values could be increased >70‐fold compared to the intermediate compound 1 (cmpd‐1, Figure 1D) leading to the advanced compounds RM‐018, RMC‐4998, and RMC‐6291.

RM‐018 was the first KRas^G12C^ molecular glue to be tested as an investigational drug in a clinical trial,^[^ ^50^ ^]^ providing a first proof of efficacy in man. Notably, RM‐018 was able to address the escape mutation KRas^G12C/Y96D^, which arises in some patients after treatment with nonglue KRas^G12C^ inhibitors such as AMG510 (Sotorasib) or MRTX849 (Adagrasib). However, the emergence of mutations in N‐Ras, B‐Raf, and MAP kinases was observed upon RM‐018 treatment which can lead to the re‐activation of Ras‐Map‐Kinase signaling.

The optimization of RMC‐4998 aimed largely to improve physicochemical and ADME parameters. This included the replacement of the phenyl ring in the macrocycle by a morpholine unit as well as the connection to the warhead. RMC‐6291 exhibited higher selectivity for KRas^G12C^ over wildtype compared to RMC‐4998, inhibited KRas^G12C^‐dependent cancer cells with an average IC_50_ of 0.11 nM,^[^ ^42^ ^]^ and led to tumor regression of xenografts in mice. Notably, RMC‐6291 seems to be also effective in Ras‐dependent RAL‐GEF and AKT‐mTOR signaling.^[^ ^42^ ^]^ RMC‐6291 is currently tested in clinical trials with a focus on lung and colorectal cancers (NCT05462717, NCT06128551, and NCT06162221). The discovery process of RMC6291 has been explained further in an article by Revolution Medicines published during the revision of this review.^[^ ^48^ ^]^

## 4 Development of RMC‐7977 and RMC‐6236

**Figure 2:**
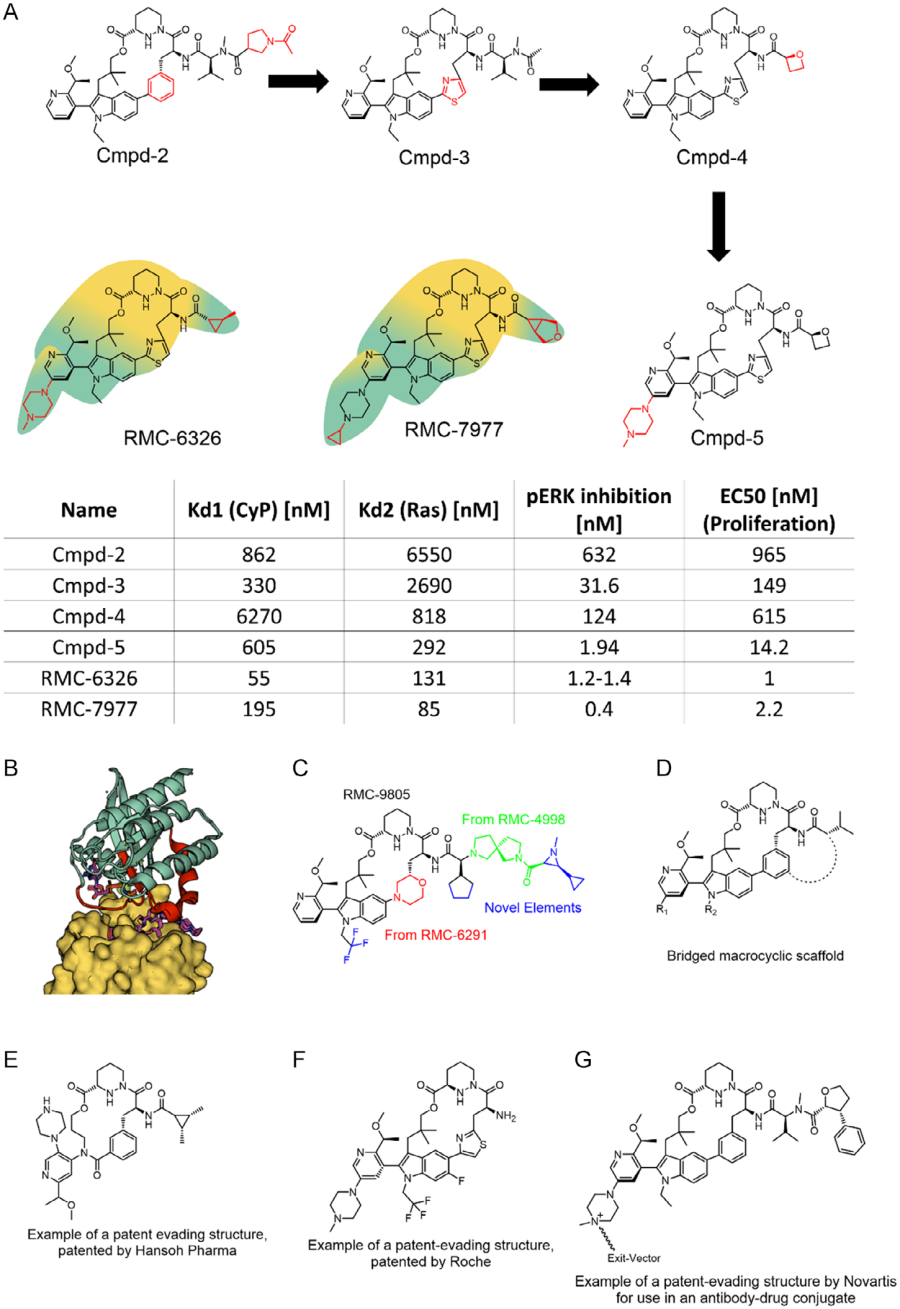
A) Ligand development of the pan‐Ras inhibitors RMC‐7977 and RMC‐6236 together with assay results. The primary CypA and KRas‐binding parts are shadowed yellow and cyan, respectively.^[^ ^51^, ^52^, ^73^ ^]^ B) Alignment of the ternary complexes between CypA, RMC‐7977 and the respective targets H‐Ras (PDB:8tbg), K‐Ras (PDB:8tbf), and N‐Ras (PDB:8tbi) with CypA colored in yellow, the Ras proteins colored in green, and the switch regions colored in red.^[^ ^51^ ^]^ C) Structure of the KRas^G12D^‐reactive molecular glue RMC‐9805.^[^ ^42^, ^57^ ^]^ Similarities to previous compounds are colored red and green, and new elements are highlighted in blue. D) Scaffold for emerging bridged macrocyclic ligands from recent patents that are inspired by the KRas‐targeting molecular glues from Revolution Medicines.^[^ ^58^ ^]^ E) A patent evading structure similar to the RMC compounds published in a patent by Hansoh Pharma.^[^ ^59^ ^]^ F) A patent‐evading structure similar to RMC‐6236 published in a patent application by Roche.^[^ ^68^ ^]^ G) A patent‐evading structure similar to the RMC compounds for the use in an antibody‐drug conjugate, published in a patent application by Novartis.^[^ ^60^ ^]^

The development of RMC‐6291 as a KRas^G12C^‐engaging molecular glue represents a landmark in molecular glue research. However, its activity relies on the presence of G12C, which is frequently found in lung cancer but less in other cancer types. This unique cysteine reactivity is not present in KRas mutants, that drive other cancer types.^[^ ^30^, ^34^ ^]^ For the development of KRas molecular glues beyond G12C, compounds based on core‐2 were crucial. Core‐2 analogs such as cmpd‐2 (**Figure** **2**A) displayed measurable CypA binding to WT KRas even in the absence of cysteine‐reactive warheads.^[^ ^51^ ^]^ Further optimization aimed to enhance the CypA‐dependent “ternary” affinity to KRas (*K*_D2_) as well as the “binary” affinity to CypA (*K*_D1_) (Figure 2A). Important modifications were the switch from the phenyl ring in the macrocycle to a thiazole (as in cmpd‐3), the replacement and optimization of the former valine moiety (as in cmpd‐4), and the introduction of a piperazine on the pyridine side chain (as in cmpd‐5). The thiazole group formed polar contacts with the side chains of the switch 1 region of K‐Ras. The replacement of the valine residue by the oxetane group improved cell permeability, bioavailability in mice, and affinity for the formation of the ternary complex with Ras, but it reduced the binary affinity of the ligand for cyclophilin A. This was regained by installing a piperazine moiety that formed additional cation‐pi interactions. The final step was gaining more hydrophobic contacts between the ligand and the proteins around the switch 2 region of KRas by introducing a cyclopropyl group at the piperazine moiety and substituting the oxetane group by a larger heterocycle which repositioned the water network favorably.

This led to the advanced pan‐Ras molecular glues RMC‐7977 and RMC‐6326, which potently inhibited several Ras‐dependent cancer cell lines such as KRas^G12V^‐dependent PDAC cells. RMC‐7977 was also able to engage other Ras subtypes beyond KRas (Figure 2B). RMC‐6236 is currently being tested in clinical phase 1 studies, alone (NCT05379985), and in combination with RMC‐6291 (NCT06128551).^[^ ^52^, ^53^ ^]^

Most remarkably, RMC‐7977 was shown not only to induce a ternary complex between CypA and KRas but to also enhance the GTPase activity of KRas in a mutant‐specific manner.^[^ ^54^ ^]^ This was most pronounced for the KRas mutant G12D and likely relied on a repositioning of the hydrolytic water‐activating amino acid network upon formation of the ternary complex. RMC‐7977 thus chemically reprogrammed CypA to act like an exogenous GTPase. The effect was also observed for NRas and HRas mutants and was reproducible for RMC6326 for all the Ras isoforms as well, although mutant‐dependent differences in mediated CypA binding were observed. Importantly, the propensity to enhance GTP hydrolysis correlated with a stronger antiproliferative efficacy in animal models.

## 5 Next‐Generation KRas‐Targeting Molecular Glues

Stimulated by the success in gluing KRas, several derivates have started to emerge. Revolution Medicines has announced RMC‐5127, which is reported as a KRas^G12V^‐selective molecular glue.^[^ ^55^, ^56^ ^]^ This is expected to translate into a favorable therapeutic window compared to pan‐Ras inhibitors. Revolution Medicines also developed RMC‐9805 (Figure 2C), which carries an aziridine moiety aimed to covalently target the carboxylate group in KRas^G12D^.^[^ ^57^ ^]^ RMC‐9805 builds on the general core‐2 structure with beneficial modifications also seen in RMC‐6291, on a rigid linker to the warhead as in RMC‐4998, and most importantly on the replacement of the cysteine‐reactive to a carboxylic acid‐reactive warhead.^[^ ^42^ ^]^

Revolution Medicines has announced additional next‐generation molecular glues such as RMC‐0708 (for Q61H) and RMC‐8839 (for G13C), the structures of which remain undisclosed. These might be linked to a series of bridged macrocyclic compounds that have been recently patented by Revolution Medicines.^[^ ^58^ ^]^ The disclosed compounds share many similarities to core‐2‐like structures but feature a second macrocycle connecting the valine to the phenyl moiety on the core‐2 macrocycle (Figure 2D). range of Ras mutations, suggesting that various Ras mutations can be addressed by tailor‐made covalent and noncovalent molecular glues.

Finally, several analogs of RMC‐6291/RMC‐6236‐like molecular glues by other biotech or pharmaceutical companies have started to appear in the patent literature. Several of these are characterized by bridging moieties as amendments to the macrocycle (Figure 2D).^[^ ^58^ ^]^ Companies such as Hansoh Pharma,^[^ ^59^ ^]^ Novartis,^[^ ^60^ ^]^ Genfleet Therapeutics,^[^ ^61^ ^]^ Nortye Biopharma,^[^ ^62^ ^]^ Nutshell Biotech,^[^ ^63^ ^]^ Medshine Discovery,^[^ ^64^ ^]^ and Roche^[^ ^65^, ^66^, ^67^, ^68^, ^69^, ^70^, ^71^, ^72^ ^]^ have filed patents of structures that are reminiscent of the ligands that Revolution Medicines developed (Figure 2E–G). Very little information on the biological activities of these compounds is available so far.

## 6 Summary and Outlook

The CypA‐assisted KRas inhibitors developed by Revolution Medicines represent a landmark in molecular glue research. They show that competitive, nondegradative molecular glues with clinical efficacy can be developed for previously undruggable targets in a rational and targeted manner. These compounds inhibit multiple Ras‐driven pathways effectively and further demonstrate that such molecular glues can have a drug‐like profile and other favorable properties compared to conventional inhibitors, for example, in overcoming escape mutations or the engagement of KRas in the on versus the off state (i.e., GTP‐ vs. GDP‐bound). Targeting KRas^on^ was claimed to be advantageous since KRas spends significantly more time in the on state in many cancer cells compared to normal cells. The recent discovery of GAP‐like activities of RMC‐7977 shows that even a gain‐of‐function can be enabled de novo by molecular glues.

Molecular glue degraders have created a lot of excitement in the field as modalities to engage otherwise undruggable targets. Nondegradative molecular glues such as those developed by Revolution Medicines are promising alternatives compared to molecular glue degraders, especially for proteins with short half‐lives that can be difficult to degrade, larger proteins where only parts of the protein should be targeted (i.e., where full degradation would be too toxic), or where specific states of the target proteins should be preferentially blocked (as shown here for KRas^on^).

In addition to providing a compelling case for the feasibility of competitive molecular glues, the findings derived from the KRas molecular glues also provide some general insights into molecular glue pharmacology:

(1) The noncovalent molecular glues demonstrated higher potency in cellular assays than in biochemical binding assays. This could be explained by the high concentration of cyclophilin A in the investigated cells. This leads to an enrichment of the compounds inside the cell and leads to an apparent higher concentration of molecular glue‐CypA complexes inside cells.^[^ ^51^ ^]^

The intracellular enrichment mediated by cyclophilin A is also consistent with a slower drug washout in the serum of the mice.^[^ ^73^ ^]^

(2) The structural analysis of the binding modes of the KRas‐engaging molecular glues show that *K*_D2_ can be enhanced by optimizing the direct contacts of the molecular glue (in complex with the presenter) with the target protein. Alternatively, the molecular glue can remodel the surface of the presenter protein so that the latter more efficiently engages the target. A similar situation has been observed for synthetic molecular glues for the model system FKBP12‐mTOR^FRB^.^[^ ^25^ ^]^

Trends in PROTAC research show that drug developers start to aim for tissue‐selective heterobifunctional degraders. This can be achieved not only by targeting specific oncogenic proteins more selectively by utilizing ternary complex formation but also by targeting cancer cell‐specific ligases or linking the drug candidates to antibodies.^[^ ^74^, ^75^ ^]^ Since target‐mediated drug disposition^[^ ^76^ ^]^ is a well‐established field with many models,^[^ ^77^ ^]^ concepts such as tissue‐specific adaptor proteins can be expected to be transferred to nondegradative molecular glues in the future.

How generally are molecular glues applicable as therapeutic modalities for challenging targets? The compounds by Revolution Medicines make an impressive case for KRas mutant/homologs, but it took a long way to get there (CypA‐based KRas^G12C^ drug discovery started at Warp Drive Bio as early as 2012). KRas^G12C^ represents a special case allowing covalent inhibitors where weak initial hits might be easier to identify. To identify chemical starting points for noncovalent molecular glues, new hit identification strategies will be important. Fragment‐based tethering approaches as pioneered by the Ottmann lab for 14‐3‐3‐based protein–protein stabilizers^[^ ^78^, ^79^, ^80^ ^]^ and presenter‐focused libraries as developed by the Liu lab^[^ ^81^, ^82^ ^]^ are intriguing approaches toward this goal.

Molecular glues will play a key role in the future to address increasingly challenging drug targets in a more refined manner. The drug candidates developed by Revolution Medicines represent an important step ahead in our capabilities toward developing these modalities.

## Conflict of Interest

The authors declare no conflict of interest.
